# Trapping all ERBB ligands decreases pancreatic lesions in a murine model of pancreatic ductal adenocarcinoma

**DOI:** 10.1002/1878-0261.13473

**Published:** 2023-07-14

**Authors:** Kathrin Hedegger, Andreas Blutke, Theresa Hommel, Kerstin E. Auer, Nishanth B. Nataraj, Moshit Lindzen, Yosef Yarden, Maik Dahlhoff

**Affiliations:** ^1^ Institute of Molecular Animal Breeding and Biotechnology, Gene Center LMU München Germany; ^2^ Institute of Veterinary Pathology, Center for Clinical Veterinary Medicine LMU München Germany; ^3^ Institute of in vivo and in vitro Models University of Veterinary Medicine Vienna Austria; ^4^ Department of Immunology and Regenerative Biology Weizmann Institute of Science Rehovot Israel; ^5^ Bugworks Research Inc, CCAMP Bengaluru India

**Keywords:** decoy molecule, EGF‐family ligands, ERBB receptors, *Kras*, mouse model, Panc‐1, PDAC

## Abstract

Pancreatic ductal adenocarcinoma (PDAC) is among the deadliest of cancers. Attempts to develop targeted therapies still need to be established. Some oncogenic mechanisms in PDAC carcinogenesis harness the EGFR/ERBB receptor family. To explore the effects on pancreatic lesions, we attempted simultaneous blockade of all ERBB ligands in a PDAC mouse model. To this end, we engineered a molecular decoy, TRAP‐F_C_, comprising the ligand‐binding domains of both EGFR and ERBB4 and able to trap all ERBB ligands. Next, we generated a transgenic mouse model (*CBA* ^*TRAP/0*^) expressing TRAP‐F_C_ ubiquitously under the control of the chicken‐beta‐actin promoter and crossed these mice with *KRAS* ^*G12D/+*^ mice (*Kras*) to generate *Trap/Kras* mice. The resulting mice displayed decreased emergence of spontaneous pancreatic lesion areas and exhibited reduced RAS activity and decreased activities of ERBBs, with the exception of ERBB4, which showed increased activity. To identify the involved receptor(s), we employed CRISPR/Cas9 DNA editing to singly delete each ERBB receptor in the human pancreatic carcinoma cell line Panc‐1. Ablation of each ERBB family member, especially the loss of EGFR or ERBB2/HER2, altered signaling downstream of the other three ERBB receptors and decreased cell proliferation, migration, and tumor growth. We conclude that simultaneously blocking the entire ERBB receptor family is therapeutically more effective than individually inhibiting only one receptor or ligand in terms of reducing pancreatic tumor burden. In summary, trapping all ERBB ligands can reduce pancreatic lesion area and RAS activity in a murine model of pancreatic adenocarcinoma; hence, it might represent a promising approach to treat PDAC in patients.

## Graphical abstract

An engineered decoy molecule, TRAP‐F_C_, was overexpressed in a mouse model for pancreatic ductal adenocarcinoma. The TRAP‐F_C_ molecule, which comprises ligand‐binding domains of both EGFR and ERBB4, is able to trap all ERBB ligands. Mice overexpressing the decoy show significantly reduced pancreatic lesions and decreased EGFR and ERBB2 activation compared with control animals.

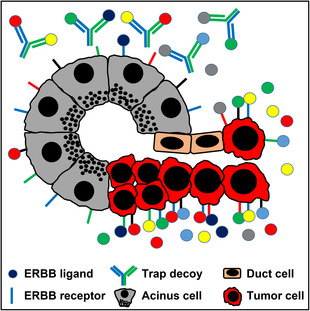

**Abbreviations**
ADM: acinar‐to‐ductal metaplasia
AREG: amphiregulin
BrdU: 5‐bromo‐2′‐deoxyuridine
BTC: betacellulin
CBA: chicken‐beta‐actin
CRISPR/Cas9: clustered regularly interspaced short palindromic repeats/CRISPR‐associated protein 9
DMEM: Dulbecco's Modified Eagle Medium
ECD: extracellular domain
EDTA: ethylenediaminetetraacetic acid
EGF: epidermal growth factor
EGFR: epidermal growth factor receptor
EPGN: epigen
EREG: epiregulin
FBS: fetal bovine serum
GAPDH: glycerinaldehyd‐3‐phosphat‐dehydrogenase
gRNA: guide RNA
HBEGF: heparin‐binding EGF‐like growth factor
ICD: intracellular domain
KO: knockout
Kras: *KRAS* ^*G12D/+*^ mice
MAB: monoclonal antibody
MAPK: mitogen‐activated protein kinase
MEK: mitogen‐activated protein kinase kinase
PanIN: pancreatic intraepithelial neoplasia
PBS: phosphate‐buffered saline
PDAC: pancreatic ductal adenocarcinoma
PFA: para‐formaldehyde
PI3K: phosphoinositide 3‐kinase
PKB: protein kinase B
RAF: rapidly accelerated fibrosarcoma
RAS: rat sarcoma
RT: room temperature
RTK: receptor tyrosine kinase
T: transgenic TRAP mice
TGFA: transforming growth factor
*Trap/Kras*
: *Ptf1a* ^*Cre/0*^; *KRAS* ^*G12D/+*^; *CBA* ^*TRAP/0*^ mice
TKI: tyrosine kinase inhibitor

## 1 Introduction

Pancreatic ductal adenocarcinoma (PDAC) is among the deadliest of all cancers in Europe and the US [1, 2]. Although more and more findings about the pathogenic mechanisms of pancreatic cancer are being revealed frequently, only few medical advances in developing therapeutic treatments exist that relevantly prolong the survival and improve patients' quality of life. There are many ongoing clinical trials investigating therapies that target aberrantly regulated proteins downstream of receptor tyrosine kinase (RTK) signaling, for example, along the rat sarcoma (RAS)/rapidly accelerated fibrosarcoma (RAF)/mitogen‐activated protein kinase kinase (MEK)/mitogen‐activated protein kinase (MAPK) or phosphoinositide 3‐kinase (PI3K)/protein kinase B (PKB/AKT) axes [3]. Although the epidermal growth factor receptor (EGFR/ERBB1/HER1) is crucial for PDAC development, the EGFR‐dependency of the tumors decreases during PDAC progression [4, 5]. EGFR belongs to the ERBB receptor family along with ERBB2 (HER2/neu), ERBB3 (HER3), and ERBB4 (HER4). We recently suggested that pan‐ERBB strategies would be more efficient than single receptor blockers since, for example, ERBB2 and ERBB4 also play compensatory roles in murine pancreatic tumorigenesis [6]. Currently, only one targeted therapy against EGFR has been approved, namely the tyrosine kinase inhibitor (TKI) erlotinib in combination with gemcitabine [7]. Nevertheless, a major problem is drug‐related toxicity, a common adverse event in monoclonal antibody (MAB) therapies and TKIs targeting the ERBB receptors, which leads to an early therapy termination [8, 9, 10]. Another promising approach is to target all ERBB ligands in order to decrease ERBB signaling, hopefully without side effects, since the decoy does not interact directly with ERBB receptors as MABs and TKIs do. The ERBB‐ligand epidermal growth factor (EGF), transforming growth factor alpha (TGFA), heparin‐binding EGF‐like growth factor (HBEGF), amphiregulin (AREG), epiregulin (EREG), and recently also betacellulin (BTC) have been implicated in pancreatic tumorigenesis [6, 11, 12, 13, 14, 15]. This group of ERBB ligands is recognized and bound by EGFR and ERBB4 and their signals are transmitted by homo‐ or heterodimerization with other ERBB receptors in order to induce intracellular signaling via the RAS/RAF/MEK/MAPK and PI3K/AKT pathways [16]. To this end, we engineered a decoy molecule, TRAP‐F_C_, by recombining the extracellular domains (ECDs) of EGFR and ERBB4, fused by a linker domain. Secretion of the decoy is ensured by the presence of a synthetic leader sequence. TRAP‐F_C_ is able to sequester all ERBB ligands, thereby inhibiting a broad range of ERBB signaling. We previously reported that after TRAP‐F_C_‐treatment the proliferation rate in the human pancreatic cancer cell lines BxPC3, Panc‐1, and MiaPaCa2 were significantly reduced and in BxPC3 cells [17], TRAP‐F_C_ even reduced proliferation after the exposure to growth factors, like EGF, TGFA, and HBEGF. Furthermore, TRAP‐F_C_ in combination with gemcitabine prolonged the survival of subcutaneously BxPC3‐injected tumors in mice [17]. In our current study, we generated transgenic mice overexpressing TRAP‐F_C_ under the control of the ubiquitous chicken‐beta‐actin promoter (*CBA* ^*TRAP/0*^) to evaluate the potential of this therapeutic approach in a genetic pancreatic cancer model. In addition, we studied the consequences of the deletion of each ERBB receptor in a human PDAC cell line. Independently, we deleted EGFR, ERBB2, ERBB3, and ERBB4 in Panc‐1 cells by using CRISPR/Cas9 gene editing, in order to analyze the dependency of Panc‐1 cells on the individual ERBB receptors in terms of ERBB and intracellular signaling, cell migration, proliferation, and RAS activity.

## 2 Materials and methods

### 2.1 Animals

All animal experiments were approved by the Committee of Animal Health and Care of the local governmental body of the state of Upper Bavaria (Regierung von Oberbayern; animal license number: ROB‐55.2‐2532.Vet_02‐19‐57), Germany, and were performed in strict compliance with the European Communities Council Directive (86/609/EEC) recommendations for the care and use of laboratory animals. Pronuclear microinjection was used to generate a mouse line that expresses the TRAP molecule consisting of the ECDs of murine EGFR and ERBB4, which are connected by a linker and a synthetic leader sequence to ensure that the molecule will be secreted. The TRAP sequence was ligated into the *EcoR*I side downstream of the chicken‐beta‐actin (CBA) promoter and rabbit‐beta‐globin splice acceptor into the expression vector pUC‐CAGGS (Addgene, Watertown, MA, USA), and upstream to the rabbit‐beta‐globin 3′‐flanking region and polyadenylation signal. The plasmid was linearized by restriction enzyme digest using *Sal*I and *Hind*III to generate the microinjection construct, which was injected into zygotes of C57BL/6N mice, which were purchased from Janvier (Le Genest St Isle, France). Thus, we obtained the *CBA* ^*TRAP0*^ mouse line ubiquitously expressing the TRAP‐decoy molecule. The genotypes of *CBA* ^*TRAP0*^ mice were verified by PCR (Qiagen, Germantown, MD, USA), employing genomic DNA from tail tips by using the oligonucleotides: pTORU‐seq: 5′‐CTACAGCTCCTGGGCAACGTG‐3′, and TRAP‐Rev: 5′‐ATCTTGCCAGTGTATAGTGTCAGC‐3′. Mice carrying floxed *KRAS* ^*G12D+*^ (*B6.129S4‐Kras* ^*tm4Tyj*^ */J*) [18] alleles or expressing Cre recombinase under the pancreas‐specific‐transcription factor 1 alpha (*Ptf1a*‐Cre; *Ptf1a* ^*tm1creHnak*^) [19] promoter have been described previously. We crossmated *Ptf1a* ^*Cre0*^; *KRAS* ^*G12D+*^ (herein referred to as *Kras*) mice with *CBA* ^*TRAP0*^ mice (herein referred to as *Trap*), herein referred to as *Trap*/*Kras* mice. In our mouse facility *Ptf1a* ^*Cre0*^; *KRAS* ^*G12D+*^ mice were crossed over 20 generations in the C57BL/6N background. Mice were maintained in the C57BL/6N background and housed under specific pathogen‐free conditions in the closed barrier facility of the Gene Center Munich at 23 °C, 50% humidity, and with a 12‐h light/dark cycle (lights on at 7 AM). They had free access to water and a standard rodent diet (V1534, Ssniff, Soest, Germany).

### 2.2 Cell culture and stimulation experiments

Panc‐1 cells (Research Resource Identifier: RRID:CVCL_0480) were purchased from CLS (Cell line service, Eppelheim, Germany) 4 months before the experiments were performed. All human permanent cell lines in the CLS cell bank were authenticated by using the STR DNA profiling analysis. Mycoplasma testing was done every 6 months using a mycoplasma detection kit (PlasmoTest, InvivoGen, Toulouse, France). The cells were cultured in Dulbecco's Modified Eagle Medium (DMEM, Merck), supplemented with 10% fetal bovine serum (FBS, Merck) and 1% Penicillin/Streptomycin (Merck, Vienna, Austria) and maintained at 37 °C and 5% CO_2_. At a confluence of 90%, cells were starved for 3 h (1% FBS) and subsequently stimulated with 25 ng·mL^−1^ of recombinant human BTC (PeproTech GmbH, Hamburg, Germany) for 10 min. Cells were then lysed in a TRIS‐based buffer (50 mm Tris, 150 mm NaCl, 1% NP40, 10% glycerol, 1 m EDTA (ethylenediaminetetraacetic acid) freshly supplemented with protease and phosphatase inhibitors (Roche) and the lysates were subjected to western blot analysis.

### 2.3 CRISPR/Cas9‐mediated *ERBB* gene editing

Guide RNA (gRNA) sequences targeting the desired loci of *EGFR*, *ERBB2*, *ERBB3*, and *ERBB4* genes were designed using benchling (Biology software, 2018; Table S1) (Benchling, San Francisco, CA, USA). Following annealing of the complementary oligonucleotides, the gRNA was cloned into the pSpCas9(BB)‐2A‐Puro (PX459) V2.0 backbone (gift from Feng Zhang, Addgene plasmid #62988, Cambridge, MA, USA) and the plasmid was transfected into Panc‐1 cells using the Lipofectamine 3000 kit (Invitrogen, Darmstadt, Germany) according to the manufacturer's instructions. After 48 h of transfection, the cells were expanded and selected using 25 μg·mL^−1^ puromycin. After 48 h, the medium was changed and single visible colonies were mechanically transferred into 96‐wells. After 100% confluence, cells were split and grown for either DNA isolation and gene‐editing verification or expansion of the verified single‐cell clones. For the assessment of gene editing, DNA of the gRNA‐targeted regions was amplified using KO‐genotyping primers (Table S2) for MiSeq (Illumina, San Diego, CA, USA) analysis. Lysis, DNA‐amplification, sample preparation, deep sequencing, and data analysis by the Outknocker webtool (www.outknocker.org) were performed as described previously [20] (miSeq primers in Table S3). Clones with all‐allelic frameshift were additionally further sub‐cloned using the NEB Kit (NEB, Frankfurt, Germany) and sequenced by Sanger (Eurofins Genomics, Ebersberg, Germany). The sequences were aligned to the designated *Ensembl* genome database; indels were verified for each *ERBB* gene locus; and the ERBB knockouts (KOs) were verified by western blot analysis. sgRNA sequences, KO‐genotyping primer sequences, miSeq primer sequences can be found in Tables S1–S3. All experiments were validated for every single ERBB knockout clone. The results depicted in this publication are represented by two representative clones per receptor knockout cell line.

### 2.4 Pancreas preparation

Male and female mice were sacrificed by cervical dislocation at the age of 4 months. The pancreas was isolated, blotted dry, and weighed to the nearest milligram. Parts of the head, tail, and central part of the pancreas were dissected, pooled, frozen on dry ice, and stored at −80 °C. The remaining tissue was fixed in 4% paraformaldehyde (PFA, in phosphate‐buffered saline, PBS, pH 7.4) overnight and subsequently embedded in paraffin for histopathological examination.

### 2.5 Histopathology and morphometric analyses

We performed a histomorphological analysis as described previously [6]. In brief, the PFA‐fixed and paraffin‐embedded pancreas of 4‐month‐old female mice was serially sectioned and every tenth section was stained with hematoxylin and eosin (H&E). The sections were then analyzed in a blinded fashion. For quantification of lesions, the slides were scanned (Axio Scan.Z1 scanner; Zeiss, Jena, Germany) and the relative areas of reactive tissue (comprising fibrosis, inflammation, acinar‐to‐ductal metaplasia (ADM), pancreatic intraepithelial neoplasia (PanIN) of grades 1–3 and PDAC) were quantified in every tenth field of vision per section by point counting [21, 22]. For this, digital images of the sampled fields of view were acquired using the netScope viewer software, and superimposed with a grid of equally spaced crosses, (Net‐Base Software GmbH, Freiburg, Germany). Crosses hitting section profiles of the respective structure were counted and related to the number of crosses hitting pancreatic tissue in all examined sections per case. On average, 800 points were counted per section. Data were analyzed by the Student's *t*‐test and plotted in graphpad prism (GraphPad Prism version 5.0 for Windows, GraphPad Software, San Diego, CA, USA).

### 2.6 RAS activity assay

To evaluate pancreatic RAS activity, the Active Ras Detection Kit (Cell Signaling, Frankfurt, Germany) was used according to the manufacturer's instructions. In brief, dissected, the frozen pancreas was homogenized or Panc‐1 cells were lysed in LBW buffer, freshly supplemented with phenylmethanesulfonyl fluoride. Panc‐1 cells were either lysed after BTC (PeproTech, Hamburg, Germany) stimulation (25 ng·mL^−1^) or unstimulated. Three hundred microgram of total lysate was incubated with the GSTRaf1Ras binding domain for 1 h at 4 °C, washed, eluted under denaturing conditions, and applied to western blot analysis detecting RAS. Total protein was analyzed by western blot analysis of the total lysates detecting total RAS and GAPDH. The amount of active RAS was measured by densitometry using imagej (NIH, Bethesda, MD, USA), referenced to total RAS and GAPDH, and plotted in graphpad prism. Data were analyzed by the Student's *t*‐test.

### 2.7 Western blot analysis

Murine pancreas was homogenized in LBW buffer (Cell Signaling), freshly supplemented with protease and phosphatase inhibitors (Roche), chilled on ice for 10 min, and centrifuged at maximum speed, and cell debris was discarded. Human cells were lysed in a TRIS‐based buffer (50 mm Tris, 150 mm NaCl, 1% NP40, 10% glycerol, 1 m EDTA; freshly supplemented with protease and phosphatase inhibitors (Roche)). Equal amounts of protein were electrophoresed on 10% SDS gels and blotted to PVDF membranes (GE Healthcare, Munich, Germany). The membranes were blocked using 5% milk and incubated in primary antibodies overnight at 4 °C. After washing, the membranes were incubated in the appropriate horseradish peroxidase‐conjugated secondary antibody at room temperature for 1 h. Immuno‐reactive bands were visualized using an ECL kit (GE Healthcare or Thermo Scientific). Antibodies and dilutions are provided in Table S4. Densitometrical analyses were performed with imagej 1.52a and plotted in graphpad prism (GraphPad Prism version 5.0). Western blots were repeated three times to confirm the shown results.

### 2.8 Hematoxylin and eosin (H&E) staining

PFA‐fixed and paraffin‐embedded pancreas tissue was sectioned and H&E‐stained (histological standard stain) using standard protocols.

### 2.9 Migration assay

For the migration assay, the ERBB‐KO clones and the control cells were seeded at 21 × 10^3^ cells per well into a 2‐well culture insert (Ibidi, Graefelfing, Germany) and allowed to adhere overnight. The insert was removed carefully, the cells were washed twice with PBS to remove debris and the media was changed. The 500 μm gap was photographed under a light microscope at the indicated time points of *n* = 3 replicates per cell clone and control, respectively. The area of the gap was measured using imagej 1.52a and plotted in graphpad prism (GraphPad Prism version 5.0 for Windows, GraphPad Software).

### 2.10 BrdU (5‐bromo‐2′‐deoxyuridine) assay

For the proliferation assay, 1 × 10^4^ cells per well were seeded in a 96‐well plate and cultured for 48 h. We performed the cell proliferation ELISA, BrdU (colorimetric) Kit (Roche, Penzberg, Germany) according to the manufacturer's instructions. In brief, the cells were labeled with BrdU for 3 h at RT and subsequently fixed by the incubation of FixDenat solution for 30 min. The cells were then incubated with a monoclonal antibody detecting BrdU, conjugated with peroxidase for 90 min at RT. Following washing, the cells were further incubated with the substrate tetramethyl‐benzidine for 20 min and the substrate reaction was stopped by adding 1 m H_2_SO_4_. The absorbance of the wells was measured at 450 nm (reference wavelength: 690 nm) in an ELISA reader. Per cell clone, four replicates were analyzed and the measurements were plotted relative to the control Panc‐1 cell line as fold change in GraphPad Prism. To evaluate the viability of the cell clones, we measured the relative number of living cells to all cells using fluidlab R‐300 (Anvajo GmbH, Dresden, Germany) and compared them to Panc‐1 control cells by plotting the fold change using graphpad prism.

### 2.11 *In vivo* orthotopic injection model

All animal experiments and methods were approved by the Weizmann Institutional Animal Care and Use Committee. Genetically modified Panc‐1 cells (RRID:CVCL_0480; ERBB1‐4 KO) were injected orthotopically into the pancreata of female 14‐week‐old CD1‐Foxn1nu mice (1 × 10^6^ cells/mouse in 20 μL of Matrigel; 1 : 1 with DMEM). Tumors were harvested 4 weeks postinjection and weight was recorded.

### 2.12 Statistics

Data are presented as mean ± SEM and compared by the two‐tailed unpaired Student's *t*‐test, and in the case of more than two groups by the analysis of variance (ANOVA) and Tukey's multiple comparison test. All data were analyzed with graphpad prism (GraphPad Prism version 5.0 for Windows, GraphPad Software). *P*‐values < 0.05 were considered statistically significant.

## 3 Results

### 3.1 ERBB receptors are less phosphorylated in *trap* mice

**Fig. 1:**
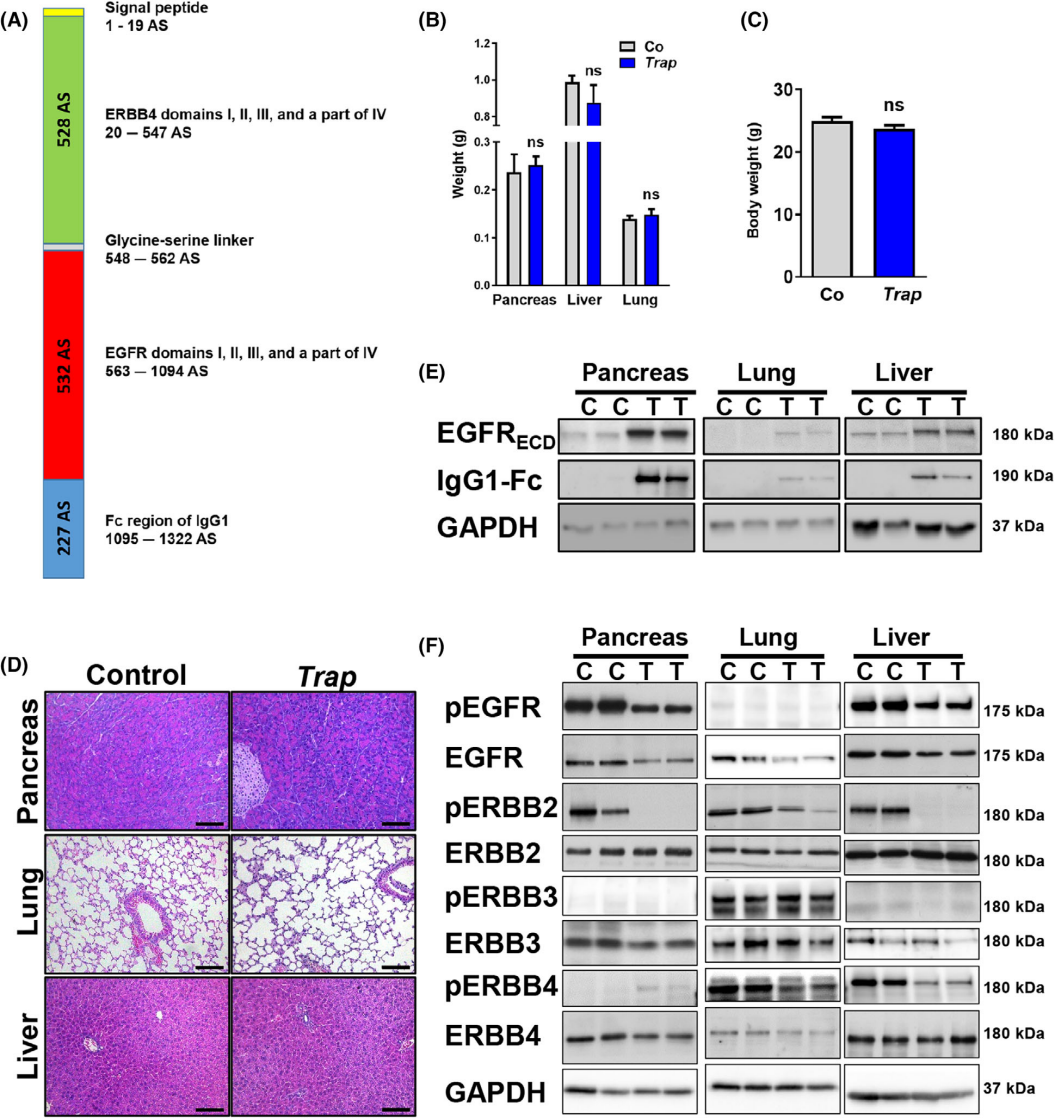
Characterization of *Trap* mice compared with control littermates. (A) Scheme of the TRAP‐Fc protein. (B) Absolute weights of pancreas, lung, and liver of *Trap* mice compared with control (Co) mice. Data were analyzed by the Student's *t*‐test and are represented as mean ± SEM (*n* = 3). (C) Body weights of *Trap* compared with control littermates, data were analyzed by the Student's *t*‐test and are represented as mean ± SEM (*n* = 3). (D) Representative H&E staining of pancreas, lung, and liver of *Trap* and control mice, scale bars, 100 μm (*n* = 4). (E) Representative western blots demonstrating the expression of TRAP by the detection of the extracellular domain (ECD) of EGFR and of the TRAP molecule in the pancreas, lung, and liver. Blots were repeated three times. C, control; T, *Trap*. (F) Representative western blots showing the phosphorylation and expression levels of the ERBB family (C‐terminal detection) in the pancreas, lung, and liver. GAPDH was used as reference protein. Blots were repeated three times. C, control; T, *Trap*.

We designed a decoy molecule (Fig. 1A), which is able to trap all 11 ERBB receptor ligands by linking the murine ECDs of the EGF‐ and ERBB4 receptor, provided it with a signal sequence to ensure its secretion, and generated a transgenic mouse line overexpressing the TRAP‐F_C_ under the control of a ubiquitous chicken‐beta‐actin (CBA) promoter. TRAP‐F_C_ transgenic mice (*Trap*) at the age of 4 months were phenotypically not distinguishable to their age‐matched control littermates, since we could not detect a difference in body and organ weight (Fig. 1B,C and Fig. S1B) and also macroscopically, as well as histologically, we revealed no differences in a variety of organs (Fig. 1D and Fig. S1C). Western blot experiments revealed that the TRAP‐F_C_ molecule was expressed in several organs of *Trap* mice (Fig. 1E and Fig. S1A) by using antibodies detecting the ECD of EGFR (EGFR_ECD_), and the IgG1‐Fc sequence of the decoy. Antibodies targeting the intracellular domain (ICD) of the ERBB receptors were used to investigate receptor expression and phosphorylation stage in pancreas, lung, and liver (Fig. 1F). As expected, phosphorylation of EGFR, ERBB2, and ERBB4 were strongly reduced in *Trap* mice, except for ERBB3. Total EGFR levels were also reduced in *Trap* mice compared with control littermates, but no alterations were detected in total ERBB2‐4 levels (Fig. 1F). However, EGFR and ERBB2 receptors were less phosphorylated in the pancreas of *Trap* mice compared with control littermates. Because EGFR and ERBB2 play an important role in PDAC we decided to crossbreed the *Trap* animals into the well‐established *KRAS* ^*G12D/+*^ PDAC mouse model to investigate the function of our decoy molecule in pancreatic cancer.

### 3.2 The TRAP molecule significantly reduces pancreatic lesion and decreases RAS activity in PDAC

**Fig. 2:**
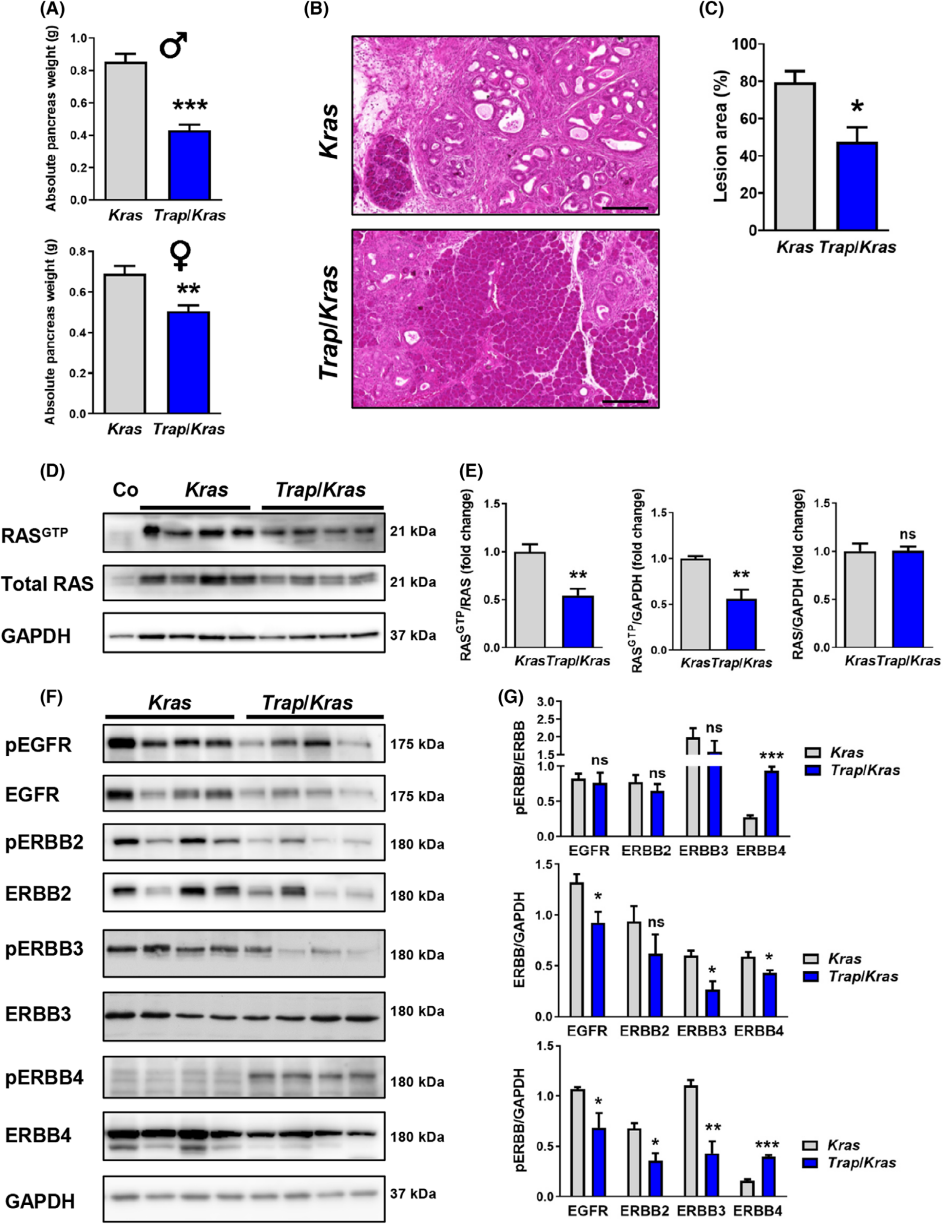
Characterization of 4‐month‐old *Trap*/*Kras* mice vs. *Kras* mice. (A) Absolute weights of the pancreas in male (upper panel) and female (lower panel) *Trap*/*Kras* and *Kras* mice (*n* = 4). (B, C) Representative H&E staining of pancreata from female mice and histopathological grading of the area of reactive tissue (i.e., fibrosis, edema, ADM, PanIN1‐3, PDAC) of *Trap*/*Kras* mice compared with age‐matched *Kras* littermates (*n* = 4). Scale bars, 200 μm. (D, E) Representative RAS activity assay and densitometric analysis of RAS activity in the pancreas of female *Trap*/*Kras* vs. *Kras* mice (*n* = 4). Blots were repeated two times. (F) Representative western blot analysis of pancreatic tissue detecting phosphorylated and total ERBB receptors of female *Trap*/*Kras* compared with *Kras* mice (*n* = 4), GAPDH served as reference protein. Blots were repeated two times. (G) Densitometrical analysis of western blot analysis (*n* = 4). Data were analyzed by the Student's *t*‐test and are represented as mean ± SEM. *P < 0.05, **P < 0.01, ***P < 0.001.

We crossed *Trap* mice in a PDAC mouse model (*Ptf1a* ^*Cre/0*^; *KRAS* ^*G12D/+*^ (*Kras*)), herein referred to as *Trap*/*Kras* mice. To compare the pancreatic lesions of *Trap*/*Kras* and *Kras* mice, the pancreatic tissue of 4‐month‐old mice was analyzed. The absolute pancreas weight of *Trap*/*Kras* mice was significantly decreased in female and male *Trap*/*Kras* animals compared with *Kras* control littermates (Fig. 2A). With a reduction of 50% in absolute pancreatic weight, the difference in male mice was more prominent than in females. The histological examination of the pancreas revealed that the pancreatic lesion area, including ADM, PanIN1‐3, and PDAC, yielded almost 80% in *Kras* mice, whereas in *Trap*/*Kras* littermates, the pancreatic lesions invaded only 50% of the pancreatic area (Fig. 2B,C). Histological examinations of other organs such as liver, lung, intestine, and kidneys did not show any metastasis (data not shown). Additionally, western blot analysis revealed that the ligands TGFA and HBEGF were significantly reduced and BTC was even abolished in the pancreas of *Trap*/*Kras* mice compared with *Kras* mice (Fig. S1D,E). A RAS activity assay demonstrated that the amount of RAS^GTP^ in *Trap*/*Kras* mice was significantly decreased compared with age‐matched *Kras* littermates (Fig. 2D,E). Since TRAP‐F_C_ captures EGFR and ERBB4 ligands and thereby the whole spectrum of ERBB ligands, we investigated ERBB receptor expression and phosphorylation by western blot analysis. Total EGFR, ERBB3, and ERBB4 expression were significantly reduced and the phosphorylation levels of EGFR, ERBB2, and ERBB3 were significantly decreased in *Trap*/*Kras* mice compared with *Kras* control littermates (Fig. 2F,G). Logically, since expression and phosphorylation levels were both decreased to a similar extent, the relative comparison of pEGFR/EGFR is unchanged (Fig. 2G). However, *Trap*/*Kras* mice show reduced EGFR, ERBB2, and ERBB3 activity compared with *Kras* control littermates. In contrast to the other ERBB receptors, the ERBB4 receptor was highly phosphorylated in the pancreas of *Trap*/*Kras* mice compared with *Kras* mice (Fig. 2F,G).

### 3.3 EGFR and ERBB2 play an important role in the signaling network of human PDAC cells

**Fig. 3:**
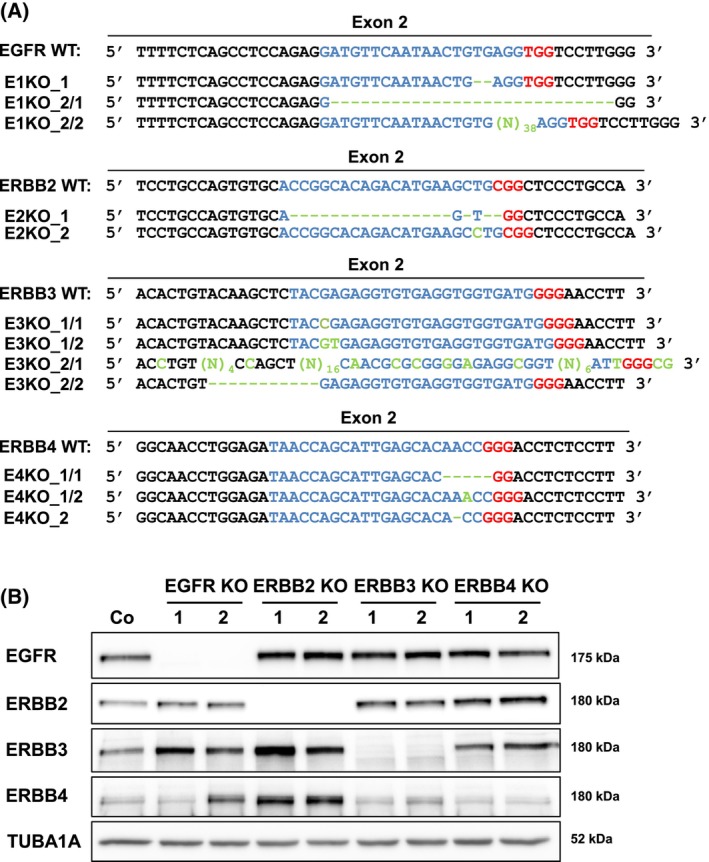
Generation and validation of ERBB‐KO Panc‐1 cells using CRISPR/Cas9 gene editing. (A) Alignment of the genetic sequence at the gRNA target site region of the designated ERBB receptors *EGFR* (E1), *ERBB2* (E2), *ERBB3* (E3), *and ERBB4* (E4) with the CRISPR/Cas9‐engineered genomic sequences of the clones EGFR‐KO C7/C9 (E1KO_1/2), ERBB2‐KO A5/B5 (E2KO_1/2), ERBB3‐KO B4/B8 (E3KO_1/2), and ERBB4‐KO B4/C9 (E4KO_1/2), blue: gRNA target sequence, red: protospacer adjacent motif, green: base pair modifications (indels). (B) Western blot analysis verifying the knockout of individual ERBB receptors in derivatives of the Panc‐1 cell line. TUB1A1 served as reference protein. Blots were repeated three times.

**Fig. 4:**
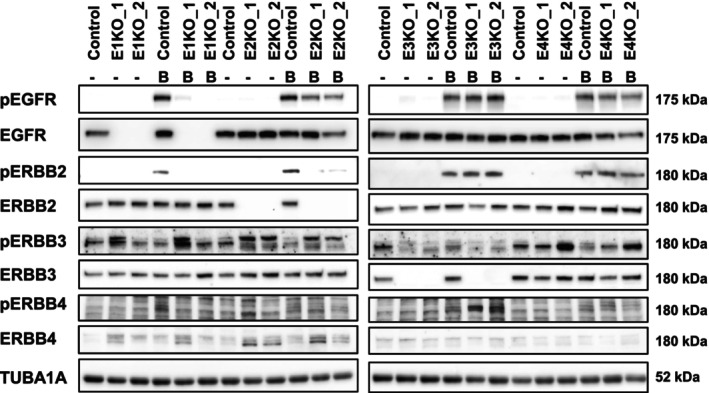
ERBB‐signaling of ERBB‐KO clones. Western blot analysis of the expression and phosphorylation state of the different ERBB receptors in two EGFR (E1), ERBB2 (E2), ERBB3 (E3), and ERBB4 (E4) knockout (KO) clones, prior (denote ‐) and following stimulation with BTC (denote B). Wild‐type Panc‐1 cells served as control. TUBA1A served as reference protein. Blots were repeated three times.

**Fig. 5:**
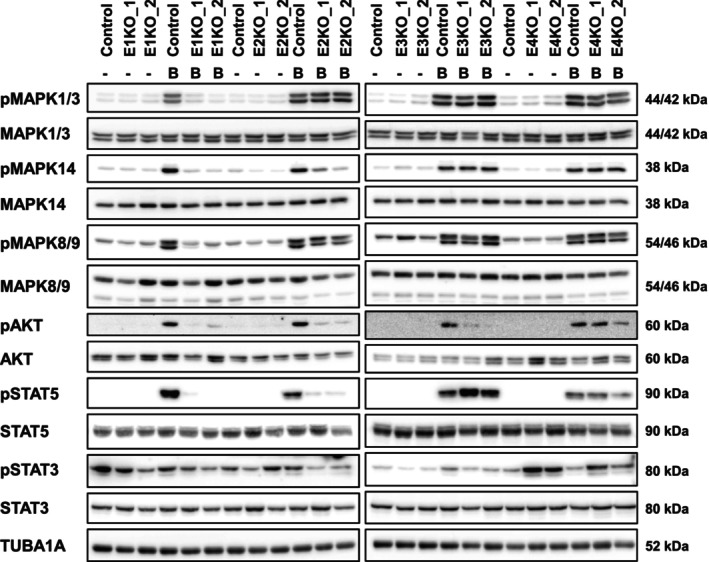
Canonical intracellular signaling of ERBB‐KO clones stimulated with betacellulin. Western blot analysis of intracellular signaling cascades in EGFR (E1), ERBB2 (E2), ERBB3 (E3), and ERBB4 (E4) knockout (KO) clones, prior (denote ‐) and following stimulation with BTC (denote B). Wild‐type Panc‐1 cells (*control*) were used in parallel. TUBA1A served as reference protein. Blots were repeated three times.

Since the expression of the ERBB ligand decoy TRAP‐F_C_ dramatically reduced the burden of premalignant lesions in *Kras* mice and highly affected the ERBB system, we set out to study the role of each ERBB receptor in a human PDAC cell line. By employing CRISPR/Cas9 gene‐editing technology we successfully deleted *EGFR*, *ERBB2*, *ERBB3*, and *ERBB4*, respectively, in the human pancreatic cancer cell line Panc‐1. For each ERBB receptor, two different knockout clones were generated, which contained an indel on each allele resulting in a frameshift (Fig. 3A). All knockout clones were verified by western blot analysis (Fig. 3B). In order to evaluate the consequences of the loss of the different ERBB genes on ERBB phosphorylation and ERBB downstream signaling cascades in the ERBB‐KO cell lines, we treated the ERBB receptor system by BTC, which is able to activate all ERBB receptors (directly or indirectly; Figs 4 and 5). While no uniformly consistent phosphorylation pattern could be detected for ERBB3 in the absence of an exogenous ligand, EGFR, ERBB2, and ERBB4 were only activated upon BTC stimulation. ERBB2 is an orphan receptor that depends on dimerization with another ERBB receptor in order to be activated, and it has been proposed that ERBB2 preferentially interacts with ERBB3 [23]. Yet, in human Panc‐1 cells deletion of ERBB3 had no impact on phosphorylation of ERBB2 upon BTC treatment. However, ERBB2 phosphorylation was absent in EGFR‐KO cells and EGFR‐phosphorylation was abolished in ERBB2 deficient cells indicating a reciprocal regulation of these receptors. Interestingly, this suggests that in human Panc‐1 cells, ERBB2 seems to form heterodimers mainly with EGFR (Fig. 4). The investigation of ERBB receptor downstream signaling the cells was activated with BTC a stimulator of all ERBB receptors and MAPK1/3 (p44/p42), MAPK8/9 (p54/p46), and MAPK14 (p38) require EGFR for activation (Fig. 5). AKT signaling needs EGFR, ERBB2, and ERBB3 activation, and the signal transducer and activator of transcription 5 (STAT5) require EGFR and ERBB2 activation. For STAT3, we could not detect a uniformly consistent phosphorylation pattern in EGFR‐KO cells, ERBB2‐KO cells, and ERBB3‐KO cells. However, the loss of ERBB4 led to an enhanced activation of STAT3 (Fig. 5).

### 3.4 Depleting individual ERBB receptors in Panc‐1 cells alters their proliferation and migration

**Fig. 6:**
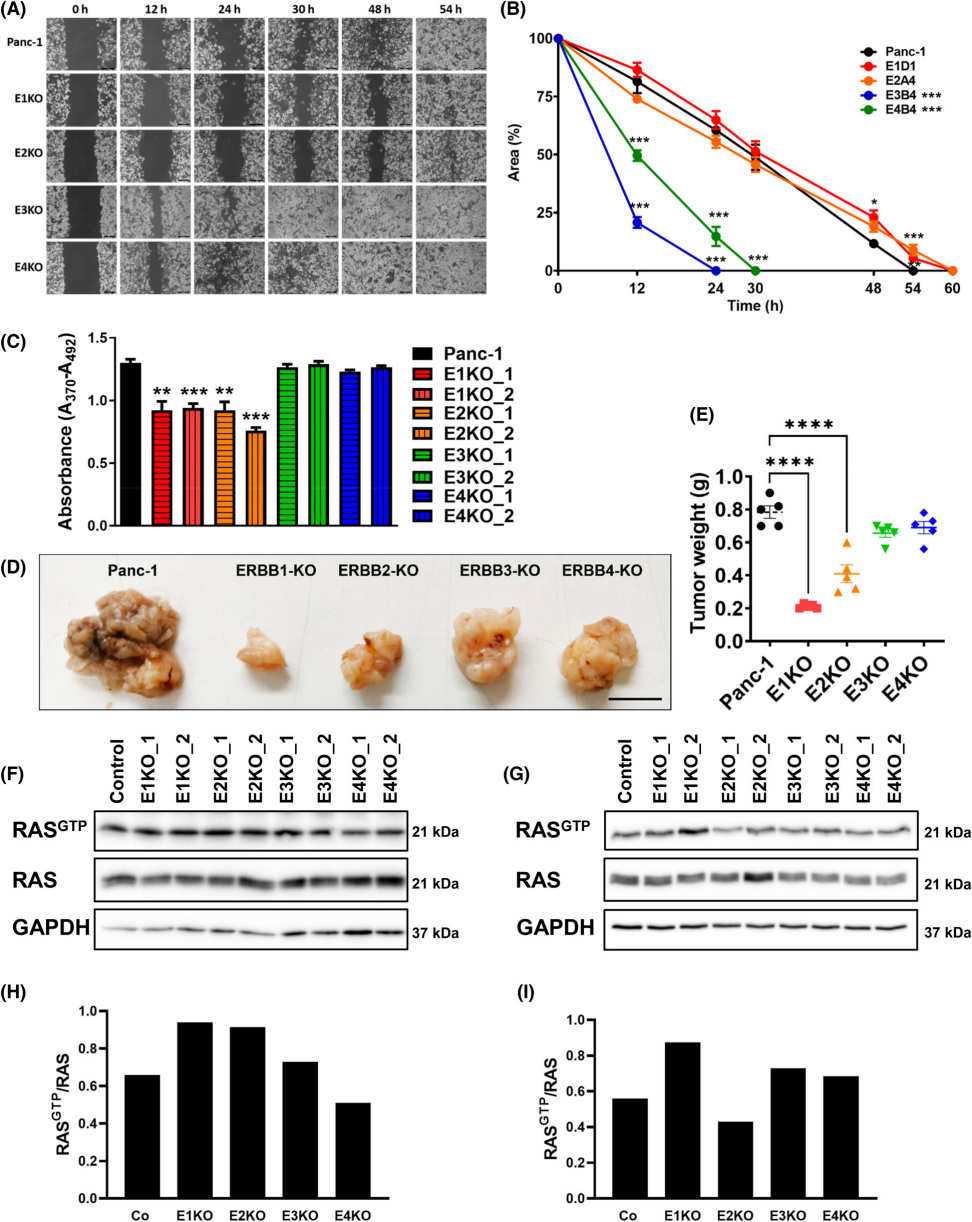
Characterization of wound closure, proliferation, and RAS activity of Panc‐1 ERBB‐KO clones. (A) Representative light‐microscopic images of Panc‐1 EGFR (E1KO_2), ERBB2 (E2KO_2), ERBB3 (E3KO_2), and ERBB4 (E4KO_2) knockout clones compared with mock Panc‐1 cells (control) at the indicated time points after wounding (*n* = 6). Scale bars, 200 μm (B) Quantitative analysis of wound closure was performed for all clones and represented as the reduction of the cell‐free area of the gap over time (*n* = 6). (C) Quantitative analysis of proliferation rate of the designated ERBB‐KO clones compared with wild‐type Panc‐1 cells (*n* = 4). (D) Picture shows representative xenograft tumors of each knockout cell line and Panc‐1 control cell line (*n* = 5). Scale bar represents 1 cm. (E) Scatter plot presents xenograft tumor weights (*n* = 5). (F, G) Representative RAS activity assay before (F) and after BTC stimulation (G). Blots were repeated two times. (H, I) Densitometrical analysis of RAS activity assay before (H) and after BTC stimulation (I) (Co: *n* = 1, E1‐4KO: *n* = 2). Data were analyzed by the Student's *t*‐test and are represented as mean ± SEM, *P < 0.05, **P < 0.01, ***P < 0.001, ****P < 0.0001.

Next, we investigated whether individual genetic ablation of each of the ERBB receptors would impact on cell migration, proliferation, tumor formation, and RAS activity in the four different ERBB knockout (KO) cell lines, compared with Panc‐1 control cells. Firstly, we performed a migration assay and revealed alterations in all ERBB‐KO cell lines compared with the MOC Panc‐1 cell line. The monolayer wound of ERBB3‐ and ERBB4‐KO clones closed significantly earlier, after 24 (ERBB4‐KO) or 30 (ERBB3‐KO) hours (Fig. 6A,B) compared with Panc‐1 control cells (48 h). Thus, both cell lines showed a much stronger migration due to the ablation of the respective receptor. By contrast, the EGFR‐KO and the ERBB2‐KO cell lines took longer to close the monolayer gap compared with the Panc‐1 control cell line. Stated differently, both cell lines migrated slower than the control cell line and showed a gap closure after 60 h, unlike the control cell line that closed the gap already after 54 h (Fig. 6A,B). Because cell proliferation is one of the main hallmarks of cancer, we employed the BrdU‐assay to comparatively monitor the proliferation of each of the four ERBB receptor‐depleted cells. Since EGFR and ERBB2 play important roles in PDAC development, it was not surprising that EGFR‐ and ERBB2‐depleted cells, unlike ERBB3‐ and ERBB4‐KO cells, showed significantly reduced proliferation rates compared with control cells (Fig. 6C). To determine whether these differences in proliferation rates translate to effects on tumorigenesis, we implanted each of the four cell lines, along with control cells, in the pancreata of CD1‐Foxn1nu mice. All animals were sacrificed 4 weeks later. As expected, the pancreata preinjected with ERBB1‐KO and ERBB2‐KO cells showed significantly reduced pancreas weight compared with the control pancreata (Fig. 6D,E). Metastases were not detected in any animal. Notably, the driver mutation of our indicator cell line, PANC‐1, is the highly frequent KRAS gene mutation in codon 12, and these cells secrete several ligands of ERBB proteins, such as the transforming growth factor alpha (TGFA). Accordingly, PANC‐1 cells respond to neither EGF nor exogenous TGFA, but an anti‐TGFA monoclonal antibody can suppress their growth [24]. Hence, we performed an *in vitro* RAS‐GTP loading assay and revealed that RAS activity is independent of all four ERBB receptors in unstimulated cells (Fig. 6F). Moreover, BTC stimulation induced no change in the amount of RAS^GTP^ in the four ERBB‐KO clones compared with the control cells (Fig. 6G).

## 4 Discussion

Pancreatic ductal adenocarcinoma is a lethal disease with a high mortality rate [1, 25] and the prognosis for patients with PDAC overall is still dire. Attempts to find an appropriate therapy often failed, although a more detailed insight into the molecular pathogenic mechanisms has been gained in the last two decades. However, based on new findings from basic research, PDAC treatment with PARP inhibitors in combination with chemotherapy seems to be promising [26, 27]. Also, EGFR in PDAC research has been of great interest because the receptor is an important player in PDAC pathogenesis [4, 5]. However, its family members ERBB2, ERBB3, and ERBB4 might also act as potent oncogenes, hence should also be considered as therapeutic targets in PDAC. It was shown that during tumorigenesis the ERBB system acts as a compensatory network, which reacts with altered expression or phosphorylation patterns following the inhibition of one member [6, 28]. This suggests that the inhibition of all ERBB receptors might be more efficient than targeting a single ERBB receptor. Nevertheless, antibody‐based therapy of cancer has achieved significant success in the last years and MABs against EGFR (cetuximab, panitumumab) [29], ERBB2 (trastuzumab, pertuzumab) [29], and ERBB3 (patritumab, seribantumab, lumretuzumab) [30] are used to treat various type of cancer. MABs against ERBB4 have been developed or are in development and will be soon brought into clinical trials; they reduce the rate of proliferation of neuregulin‐1‐dependent breast cancer cells *in vivo* [31]. Antibody‐based therapy for cancer has become important in the last decade to support chemotherapy or immunotherapy. Unfortunately, skin toxicity is a common drug‐related adverse event in cancer patients treated with ERBB MABs and with TKIs. Many cancer patients (45–100%) suffer from the toxic side effects of the MABs and terminate the therapy too early [8, 9, 10]. An EGFR‐MAB therapy may be associated with strong dermatological toxicities, inflammations, *Staphylococcus aureus* superinfection, and hematopoietic side effects such as papulopustular rush, nail inflammation, dry skin, alopecia, increased growth of eyelashes and facial hair, and strong pruritus [9]. Similar toxicity effects are described in therapies with EGFR‐TKIs (e.g., afatinib, erlotinib), which are often associated with refractory pruritus and diarrhea [32, 33]. These events are often triggers for psycho‐social effects, which decrease the patients' quality of life. Patients treated with trastuzumab or pertuzumab often suffer from infected skin and nails, esophagitis and pneumonitis [34, 35] and from heart dysfunction and strong diarrhea [36]. Patients treated with lumretuzumab suffer from diarrhea, fatigue, decreased appetite, as well as from rash, and dry skin [30, 37]. A novel strategy to reduce ERBB‐signaling and to simultaneously avoid toxicity lies in the possibility to target the ERBB receptor ligands. Our decoy molecule combines the ligand‐binding domains of EGFR and ERBB4 and is therefore able to sequester all ERBB ligands, thereby possibly decreasing ERBB signaling. Inhibition of the ligands instead of the receptors could be an effective alternative treatment because it was shown that the overexpression of EGFR ligands, for example, TGFA, correlates with poor survival in PDAC [11], colorectal cancer [38], bladder cancer [39] or head and neck cancer [40]. Since the decoy acts indirectly on the receptors and not directly like MABs and TKIs, there might be fewer toxicity effects, while the reduction of the ligands inhibits tumor growth as described in the ligand knockout mouse models. However, it is notable that no decoy approach has been tested in cancer patients, hence potential adverse effects remain unknown. To evaluate the decoy in mice, we crossed TRAP‐F_C_ mice in the *KRAS* ^*G12D/+*^ PDAC mouse model and revealed that *Trap*/*Kras* mice acquired reduced pancreatic lesions, possibly due to a decreased RAS activity compared with age‐matched *Kras* controls. Thus, along with efficacy tests, our animal model would permit in‐depth, long‐term exploration of side effects of the decoy molecule. It is noteworthy that so far we have followed our *Trap* animals for 6 months but have observed no overt toxicity. Our results indicate that TRAP‐F_C_ is able to reduce the pancreatic lesion area without showing any detectable toxicity. All ERBB receptors were significantly less expressed in the pancreas of *Trap*/*Kras* mice, except for ERBB3. As expected, the phosphorylation state of EGFR, ERBB2, and ERBB3 was significantly reduced. These data, altogether, point to a reduction in pan‐ERBB signaling. In our recent publication, we have shown that the genetic deletion of a single ligand of the ERBB family, BTC, also led to a reduced tumor burden in *Kras* mice, which was due to decreased EGFR‐signaling [6]. Another group associated the inhibition of AREG with the inhibition of EGFR/ERBB3‐heterodimer formation and a reduction in MAPK1/3 and AKT signaling in AsPC‐1 cells [41]. Both studies emphasized the power of ERBB ligands in pancreatic cancer. Loss of BTC [42], EGF [43], and EPGN [44] shows no phenotype in mice, but the deletion of TGFA [45, 46], HBEGF [42], EREG [47], and AREG [48] leads to severe alteration such as poorly differentiated lungs and enlarged dysfunctional hearts, gastric mucosal lesions, chronic dermatitis, curly coat of hair and growth restriction. However, none of these symptoms were observed in *Trap* mice. As we have shown by western blot, in trap mice are the levels of ERBB ligands TGFA and EGF only reduced and not deleted, but this seems to be sufficient enough to reduce tumor growth and does not cause side effects. However, ERBB4 phosphorylation was increased, indicating a reciprocal regulation in *Trap*/*Kras* compared with *Kras* mice, which does not fit the remaining results. The ERBB4 receptor has many faces and it turned out that its function has to be looked at in a context‐dependent manner. First, the affinity of ERBB4 ligands to the ERBB4‐TRAP domain might be weaker than to the physiologic receptor so that some ligands might bind and activate the wild‐type ERBB4 receptor in the presence of TRAP‐Fc. Second, ERBB4 might act autonomously in response to the down‐regulation of the remaining receptors, as we have observed previously in *Kras* mice lacking EGFR [6] or as revealed in breast cancer cells, where the intracellular domain (IDC80) of ERBB4 stands in against the tyrosine kinase inhibition of other ERBB members in order to maintain oncogenic signaling [28]. However, it is notable that we have not observed ICD activation of ERBB4 in our experimental system. Third, ERBB4 phosphorylation might be considered a positive way to protect *Kras* mice from developing PDAC. Indeed, ERBB4 might have tumor‐suppressive functions in different cancers and its role in cancer remains controversial [49]. On the other hand, its activation could also be associated with a protective effect since ERBB4 phosphorylation plays a protective role in acute pancreatitis [50]. Hence, the question remains whether ERBB4 has a tumor‐suppressive function or whether this receptor is the main driver of tumor development in *Trap*/*Kras* mice after signaling by the three other ERBB members was silenced through the inhibition of their ligands.

In our *in vivo* PDAC animal model, all four ERBB receptors should be equally affected by the TRAP decoy. To investigate, which receptor affects the tumor cells the most in PDAC, we chose a human *in vitro* model, as it is easier to analyze the signaling pathways of each receptor in cell culture. To evaluate ERBB receptor dependencies of PDAC in human cells, we deleted each single ERBB receptor using CRISPR/Cas9 technology in Panc‐1 cells and investigated the activation of the ERBB signaling network. We found that ERBB2 activation was highly dependent on the presence of EGFR. On the contrary, EGFR activation was partially dependent on ERBB2 and also, but to a lesser extent, dependent on ERBB4. ERBB4 expression was increased in ERBB2‐KOs, which indicates that these three receptors share a co‐dependency upon BTC stimulation, which is in line with what has previously been observed in a murine PDAC model [6]. However, the influence of ERBB4 on canonical intracellular signaling is negligible since changes in MAPK signaling were only affected by mainly EGFR and to a lesser extent by ERBB2. MAPK1/3 signaling is uniquely induced by EGFR and our knockout experiments in mice have shown that upon BTC stimulation, the ERBB4 receptor highly influences EGFR, MAPK1/3, and MAPK8/9 signaling [6]. By contrast, EGFR, ERBB2, and ERBB3 were similarly involved in AKT signaling in Panc‐1 cells, while no change in AKT signaling has been observed in our murine KO models. However, the only compensatory mechanism on the receptor level was observed by the upregulation of ERBB4 expression upon the loss of ERBB2. Intracellularly, we also found compensatory STAT3 phosphorylation upon the loss of ERBB4. We found the compensation of ERBB3 signaling by STAT5 activation after BTC stimulation. However, STAT3 is constitutively activated in Panc‐1 cells [51] and is independent of EGFR, ERBB2, or ERBB3 activation. This is not surprising since STAT3 is a component of the receptor‐associated Janus kinases (JAK) pathway mediating cytokine signaling. STAT3 could compensate for the loss of ERBB4 signaling, and since STAT3 has been shown to be required for PDAC development and acts also as a driver of cell proliferation in PDAC [52], it might be the reason for the slightly enhanced proliferation rates we observed in ERBB4‐KO cells. These results indicate a crosstalk of the ERBB receptors with the JAK/STAT pathway. However, many pathway downstream of the ERBB receptors such as MAPK, AKT, JAK/STAT, or RAS could also be activated by other RTKs like hepatocyte growth factor receptor (MET), fibroblast growth factor receptor (FGFR), platelet‐derived growth factor receptor alpha (PDGFRA), or receptor protein‐tyrosine kinase (AXL), which are often associated with ERBB receptors and could be influenced by ERBB inhibitors [53].

Loss of EGFR or ERBB2 in human Panc‐1 cells results in delayed migration, reduced proliferation rate, and increased tumor growth behavior. Just an increased proliferation rate and also an increased migration are important hallmarks of cancer [54]. The wound healing assay used for cell migration behavior also reflects proliferation rate why the results from the proliferation assays are also reflected in this test. However, our results show that both EGFR and ERBB2 play a significant role in PDAC and their loss leads to slowed tumor growth. We have shown the importance of EGFR and ERBB2 for PDAC in several previous studies and it has also been demonstrated by other groups [4, 5, 6]. Surprisingly, ERBB3‐ and ERBB4‐KO cells show a completely different behavior, no changes in proliferation and tumor growth, and even accelerated migration. An opposite behavior in ERBB3 and ERBB4‐KO cells is not strongly surprising and could be a counter‐regulatory behavior of the ERBB network. Although many intracellular signaling pathways were reduced in the ERBB‐KO clones, we could not detect a reduction of RAS activity in each single ERBB receptor. However, particularly an unaltered RAS activation is interesting, since we found a strong reduction in MAP kinase signaling in EGFR‐KO clones. It is intriguing that this reduction is not linked to reduced RAS activity, since RAS acts upstream of MAPK. In a previous study, we correlated the loss of EGFR in a pancreatic cancer mouse model to a significantly reduced amount of RAS^GTP^ molecules [6]. Possibly, RAF activation might play a role in this complex signaling mechanism and should be investigated in more detail. It might also be possible, that the loss of a single receptor is not sufficient to reduce RAS activity. Likely RAS activity is only induced upon the heterodimerization of the ERBB receptors and we identified a co‐dependency of at least EGFR and ERBB2. Simultaneous inhibition of these two receptors might unveil more insights into the complex function of the ERBB network.

## 5 Conclusion

Since the inhibition of a single ERBB receptor seems insufficiently effective in treating pancreatic adenocarcinoma, our study provides evidence that inhibiting pan‐ERBB‐signaling by trapping all ERBB ligands is a more promising approach. In line with this concept, anti‐tumorigenic effects of pan‐ERBB have been demonstrated already in pancreatic cancer cell lines [17]. Our study takes this one step further by using an *in vivo* model and demonstrating that an ERBB ligand trap reduced pancreatic lesion area in mice. This study and our recently published work emphasize the urgency of using pan‐ERBB strategies instead of blocking single receptors of the family. Unfortunately, a recent study showed that afatinib, a TKI that inhibits EGFR and ERBB2, in combination with the cytostatic drug gemcitabine, did not improve gemcitabine treatment alone [55]. The new high efficacy of pharmacological strategies utilizing drug combinations as it is used in HIV therapy could also be useful in cancer treatment. A combination of chemotherapy with different MAPs and TKIs together with decoy molecules may be useful.

## Conflict of interest

The authors declare no conflict of interest.

## Author contributions

MD, KH, and YY involved in the study concept and design. MD and KH involved in the acquisition of data. MD, KH, ML, AB, TH, and NBN involved in the analysis and interpretation of data. MD, KH, and YY involved in drafting of the manuscript. MD, KH, ML, and YY involved in the critical revision of the manuscript for important intellectual content. MD, KH, ML, and KEA involved in statistical analysis. Obtained funding was not applicable. MD, KH, YY, ML, AB, TH, and NBN involved in administrative, technical, or material support. MD and YY involved in study supervision.

### Peer review

The peer review history for this article is available at https://www.webofscience.com/api/gateway/wos/peer-review/10.1002/1878-0261.13473.

## Supporting information


**Fig. S1.** Expression of TRAP‐F_C_ in transgenic mice.

**Table S1.** Guide RNA‐design for *ERBB*‐KOs.
**Table S2.** Primers used for *ERBB*‐KO‐genotyping.
**Table S3.** Primers used for miSeq.
**Table S4.** Antibodies used for western blot analysis.

## Data Availability

All data generated or analyzed during this study are included in this published article and its supplementary information files.
