# Supplementary material for: Trapping all ERBB ligands decreases pancreatic lesions in a murine model of pancreatic ductal adenocarcinoma
Source: Mol Oncol. 2023 Jul 14;17(11):2415–31. doi: 10.1002/1878-0261.13473 (PMC10620123; doi:10.1002/1878-0261.13473)
Supplement: Supplementary file 1 — Fig. S1. Expression of TRAP‐FC in transgenic mice. [file MOL2-17-2415-s002.pdf]

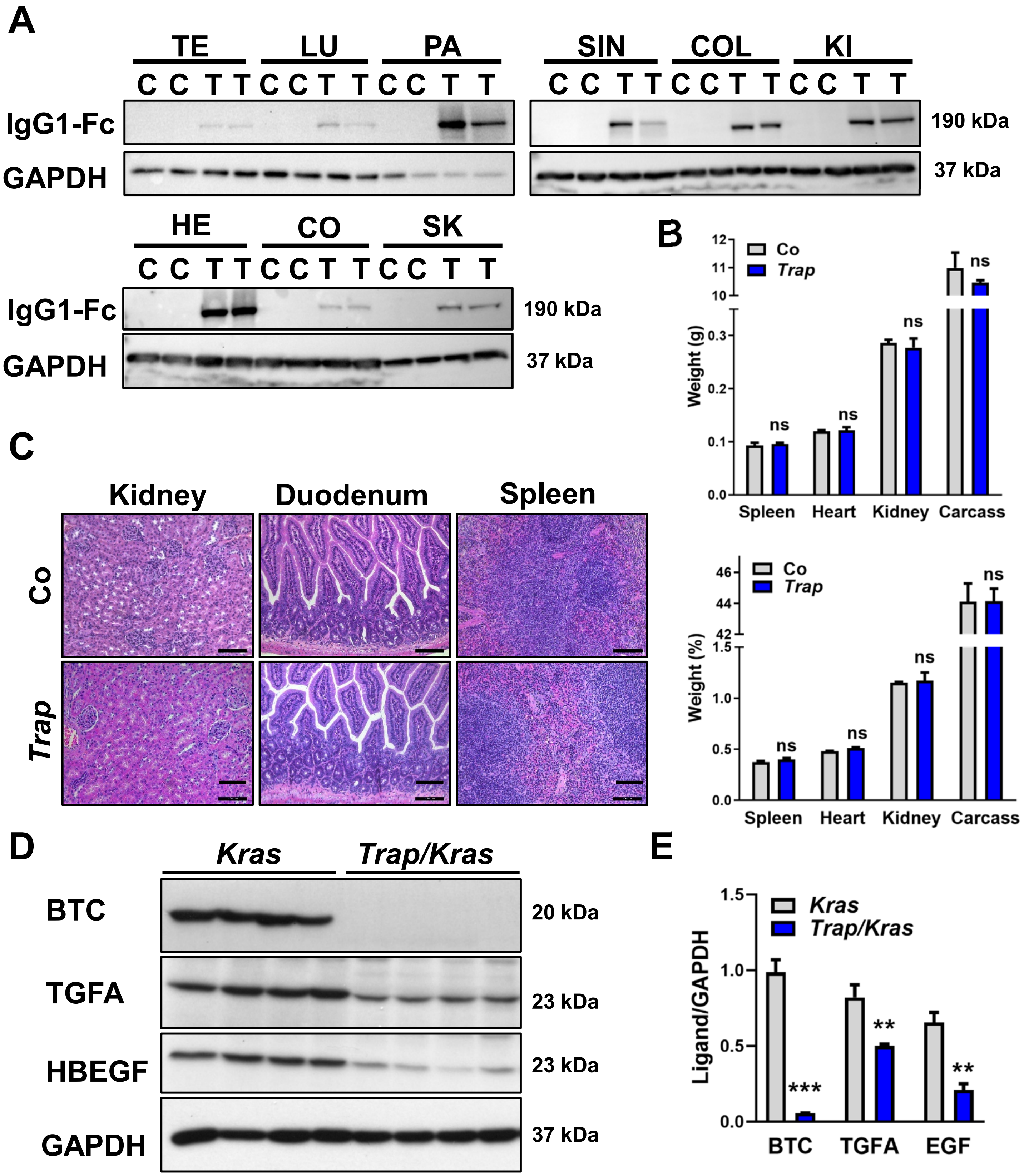

Supplementary Figure S1: (A) TRAP-F_C is expressed in several tissues of *Trap* transgenic mice. Western blots reveal the TRAP-F_C protein in testis (TE), lung (LU), pancreas (PA), small intestine (SIN), colon (COL), kidney (KI), heart (HE), cortex (CO), and skin (SK) of transgenic animals (T) compared to controls (C). GAPDH was used as reference protein. (B) Total and relative (to body weight) weight of organs and carcass of *Trap* mice compared to control mice (n=3). As statistical analysis, a Student`s *t*-test was performed. (C) Representative H&E stainings of kidney, duodenum and spleen of *Trap* mice compared to their age-matched controls, scale bars: 100 µm. (D) Western blot analysis of BTC, TGFA, and HBEGF expression in the pancreas of *Trap*/*Kras* animals compared to *Kras* mice. GAPDH was used as reference protein. (E) Densitometric analysis of ligand expression (n=4). Data are presented as mean±SEM and analyzed by Student`s *t*-test. ***P*<0.01, ****P*<0.001.

Hedegger et al., Figure S1
