# Supplementary material for: Trapping all ERBB ligands decreases pancreatic lesions in a murine model of pancreatic ductal adenocarcinoma
Source: Mol Oncol. 2023 Jul 14;17(11):2415–31. doi: 10.1002/1878-0261.13473 (PMC10620123; doi:10.1002/1878-0261.13473)
Supplement: Supplementary file 2 — Table S1. Guide RNA‐design for ERBB‐KOs. Table S2. Primers used for ERBB‐KO‐genotyping. Table S3. Primers used for miSeq. Table S4. Antibodies used for western blot analysis. [file MOL2-17-2415-s001.docx]

Table S1. Primers used for *ERBB* KO-genotyping.

| hEGFR (5‘-forward-3‘) | TCACTAAGTTAGTAAGCTTTGC |
| --- | --- |
| hEGFR (5‘-reverse-3‘) | CTCTGTAAGACTTGTCATTGC |
| hERBB2 (5‘-forward-3‘) | GTGGCATGACTTGGAGTGAG |
| hERBB2 (5‘-reverse-3‘) | ATCCAGACATGACCTCGGC |
| hERBB3 (5‘-forward-3‘) | AGGCCACTACAGCTTCTGC |
| hERBB3 (5‘-reverse-3‘) | AGGAAGGGTGAACTCCAGC |
| hERBB4 (5‘-forward-3‘) | GGTCTATGCCTCTTAATGACG |
| hERBB4 (5‘-reverse-3‘) | TGACTCTCCTAGTTCACAGC |

Table S2. Guide RNA-design for *ERBB* KOs.

| hEGFR_gRNA (5‘-forward-3‘) | CACCGGATGTTCAATAACTGTGAGG |
| --- | --- |
| hEGFR_gRNA (5‘-reverse-3‘) | aaacCCTCACAGTTATTGAACATCc |
| hERBB2_gRNA (5‘-forward-3‘) | CACCGACCGGCACAGACATGAAGCTG |
| hERBB2_gRNA (5‘-reverse-3‘) | aaacCAGCTTCATGTCTGTGCCGGTc |
| hERBB3_gRNA (5‘-forward-3‘) | CACCGACACTGTACAAGCTCTACGAG |
| hERBB3_gRNA (5‘-reverse-3‘) | aaacCTCGTAGAGCTTGTACAGTGTc |
| hERBB4_gRNA (5‘-forward-3‘) | CACCGTAACCAGCATTGAGCACAACC |
| hERBB4_gRNA (5‘-reverse-3‘) | aaacGGTTGTGCTCAATGCTGGTTAc |

Table S3. Primers used for miSeq.

| hEGFR (5‘-forward-3‘) | ACACTCTTTCCCTACACGACGctcttccgatctGCCAAGGCACGAGTAACAAGC |
| --- | --- |
| hEGFR (5‘-reverse-3‘) | TGACTGGAGTTCAGACGTGTGctcttccgatctGCTCTGTAAGACTTGTCATTGCC |
| hERBB2 (5‘-forward-3‘) | ACACTCTTTCCCTACACGACGctcttccgatctTTCTCCCTGTCTGAGGTGGC |
| hERBB2 (5‘-reverse-3‘) | TGACTGGAGTTCAGACGTGTGctcttccgatctGCAGGAAGGACAGGCTGGC |
| hERBB3 (5‘-forward-3‘) | ACACTCTTTCCCTACACGACGctcttccgatctGCTGAGAATTTGTGTCCAGCC |
| hERBB3 (5‘-reverse-3‘) | TGACTGGAGTTCAGACGTGTGctcttccgatctCTAGGGGAGAATAAAGAGGAGC |
| hERBB4 (5‘-forward-3‘) | ACACTCTTTCCCTACACGACGctcttccgatctCGTGTATCTTCTTGTCTTTGCAG |
| hERBB4 (5‘-reverse-3‘) | TGACTGGAGTTCAGACGTGTGctcttccgatctGCACACAGGTCTGCCTGTATG |

Table S4. Antibodies used for Western blot analysis.

| Antigen | Antibody | Host | Dilution |
| --- | --- | --- | --- |
| p-EGFR (Tyr1068) | Cell Signaling, Boston, USA #3777 | Rabbit | 1:1000 |
| p-ERBB2 (Tyr877) | Cell Signaling #2241 | Rabbit | 1:1000 |
| p-ERBB3 (Tyr1289) | Cell Signaling #4791 | Rabbit | 1:1000 |
| p-ERBB4 (Tyr 1258) | Abcam, Cambridge, UK ab76132 | Rabbit | 1:1000 |
| EGFR | Santa Cruz, Heidelberg, Germany #03 | Rabbit | 1:500 |
| ERBB2 | Santa Cruz #284 | Rabbit | 1:500 |
| ERBB3 | Santa Cruz #285 | Rabbit | 1:500 |
| ERBB4 | Santa Cruz #283 | Rabbit | 1:500 |
| p-p46/54 MAPK (p-SAPK/JNK, p-MAPK8/9) | Cell Signaling #9251 | Rabbit | 1:1000 |
| p46/54 MAPK (SAPK/JNK,  p-MAPK8/9) | Cell Signaling #9252 | Rabbit | 1:1000 |
| p-p44/42 (p-MAPK1/3,  p-ERK1/2) | Cell Signaling #4370 | Rabbit | 1:1000 |
| p44/42 MAPK (MAPK1/3, ERK1/2) | Cell Signaling #9102 | Rabbit | 1:1000 |
| p-p38 MAPK (p-MAPK14) | Cell Signaling #9211 | Rabbit | 1:1000 |
| P38 (MAPK14) | Cell Signaling #9212 | Rabbit | 1:1000 |
| p-AKT/PKB | Cell Signaling #9271 | Rabbit | 1:1000 |
| AKT/PKB | Cell Signaling #9272 | Rabbit | 1:1000 |
| p-STAT5 | Cell Signaling #9359 | Rabbit | 1:1000 |
| STAT5 | Cell Signaling # | Rabbit | 1:1000 |
| p-STAT3 | Cell Signaling #9145 | Rabbit | 1:1000 |
| STAT3 | Cell Signaling #12640 | Rabbit | 1:1000 |
| RAS | Cell Signaling #8832 | Mouse | 1:200 |
| BTC | R&D Systems #AF1025 | Goat | 1:1000 |
| TGFA | LS Bio #LS-B14370 | Rabbit | 1:1000 |
| HBEGF | Santa Cruz #SC-365182 | Mouse | 1:1000 |
| TUBA1A | Cell Signaling, # 2125 | Rabbit | 1:5000 |
| GAPDH | Cell Signaling, #2118 | Rabbit | 1:5000 |
| Goat α Rabbit | Cell Signaling, #7074 | Goat | 1:2500 |
| Rabbit α Mouse | Cell Signaling, #7076 | Rabbit | 1:2000 |
| Donkey α Goat | R&D Systems #HAF109 | Donkey | 1:5000 |
